# Do antibody–drug conjugates increase the risk of sepsis in cancer patients? A pharmacovigilance study

**DOI:** 10.3389/fphar.2022.967017

**Published:** 2022-11-09

**Authors:** Shuang Xia, Yi-Chang Zhao, Lin Guo, Hui Gong, Yi-Kun Wang, Rui Ma, Bi-Kui Zhang, Yue Sheng, Mayur Sarangdhar, Yoshihiro Noguchi, Miao Yan

**Affiliations:** ^1^ Department of Pharmacy, The Second Xiangya Hospital, Central South University, Changsha, China; ^2^ International Research Center for Precision Medicine, Transformative Technology and Software Services, Hunan, China; ^3^ Toxicology Counseling Center of Hunan Province (TCCH), Changsha, China; ^4^ Department of Hematology, The Second Xiangya Hospital, Central South University, Changsha, China; ^5^ Division of Biomedical Informatics, Cincinnati Children’s Hospital Medical Center, Cincinnati, OH, United States; ^6^ Division of Oncology, Cincinnati Children’s Hospital Medical Center, Cincinnati, OH, United States; ^7^ Department of Pediatrics, University of Cincinnati College of Medicine, Cincinnati, OH, United States; ^8^ Laboratory of Clinical Pharmacy, Gifu Pharmaceutical University, Gifu, Japan

**Keywords:** antibody–drug conjugates, sepsis, pharmacovigilance, FAERS, data mining

## Abstract

**Introduction:** Antibody–drug conjugates (ADCs) produce unparalleled efficacy in refractory neoplasms but can also lead to serious toxicities. Although ADC-related sepsis has been reported, the clinical features are not well characterized in real-world studies.

**Objective:** The aim of this study was to identify the association between ADCs and sepsis using FAERS data and uncover the clinical characteristics of ADC-related sepsis.

**Methods:** We performed disproportionality analysis using FAERS data and compared rates of sepsis in cancer patients receiving ADCs vs. other regimens. Associations between ADCs and sepsis were assessed using reporting odds ratios (RORs) and information component (IC). For each treatment group, we detected drug interaction signals, and conducted subgroup analyses (age, gender, and regimens) and sensitivity analyses.

**Results:** A total of 24,618 cases were reported with ADCs between Q1, 2004 and Q3, 2021. Sepsis, septic shock, multiple organ dysfunction syndrome, and other sepsis-related toxicities were significantly associated with ADCs than other drugs in this database. Sepsis and multiple organ dysfunction syndrome have the highest safety concerns with ADCs compared with other anticancer monotherapies. Gemtuzumab ozogamicin and inotuzumab ozogamicin showed increased safety risks than other ADCs. For the top nine ADC-related sepsis, males showed higher sepsis safety concern than females (*p* <0.001); however, age did not exert influence on the risk of sepsis. We identified that 973 of 2,441 (39.9%) cases had acute myeloid leukemia (AML), and 766 of 2613 (29.3%) cases on ADCs died during therapy. Time-to-onset analysis indicated ADC-related sepsis is prone to occur within a month after administration. Co-administration of ADCs with colony-stimulating factors, proton pump inhibitors, H2-receptor antagonists, or CYP3A4/5 inhibitors showed to synergistically increase the risk of sepsis-related toxicities.

**Conclusion:** Antibody–drug conjugates may increase the risk of sepsis in cancer patients, leading to high mortality. Further studies are warranted to characterize the underlying mechanisms and design preventive measures for ADC-related sepsis.

## Introduction

Antibody–drug conjugates (ADCs) are a relatively new class of anticancer agents designed to merge the selectivity of monoclonal antibodies with cell-killing properties of chemotherapy. They are commonly described as the “Trojan horses” of therapeutic armamentarium because of their capability of directly conveying cytotoxic drug (payloads) into the tumor space, thus transforming chemotherapy into a targeted agent (Criscitiello et al., 2021). The FDA has approved 12 ADCs, which could be categorized by different kinds of payload, tubulin polymerization inhibitors (trastuzumab emtansine, enfortumab vedotin, brentuximab vedotin, polatuzumab vedotin, belantamab mafodotin, and tisotumab vedotin), DNA-damaging agents (gemtuzumab ozogamicin, inotuzumab ozogamicin, trastuzumab deruxtecan, and sacituzumab govitecan), pyrrolobenzodiazepine (loncastuximab tesirine), and truncated exotoxin (moxetumomab pasudotox). ADCs have an excellent risk-to-benefit ratio (Chau et al., 2019) in many types of neoplasms and seem suited to provide benefit for patients with treatment-refractory cancers (Drago et al., 2021). A recent study indicated that grade 3/4 anemia, neutropenia, and peripheral neuropathy were consistently reported for ADCs whose payload is monomethyl auristatin E (MMAE), thrombocytopenia and hepatic toxicity for emtansine (DM1), and ocular toxicity for monomethyl auristatin F (MMAF) (Masters et al., 2018). Another study showed that despite the use of antibodies targeting antigens abundantly and exclusively expressed on cancer cells (i.e., target cells), dose-limiting toxicities (DLTs) in normal cells/tissues are frequently reported even at suboptimal therapeutic doses (Mahalingaiah et al., 2019).

Sepsis is a condition that is associated with extremely high mortality and, for many of those who survive, severe morbidity. Cancer patients with sepsis have higher mortality rates than non-cancer patients (Hensley et al., 2019; Manjappachar et al., 2022). A recent study further indicated that septic shock in patients with hematologic malignancies is associated with a high mortality rate and poor 90-day survival compared with the control group. The World Health Organization (WHO) designated sepsis a global health priority in 2017 and adopted a resolution to improve the prevention, diagnosis, and management of sepsis (Cecconi et al., 2018).

The first case of sepsis was reported with brentuximab vedotin in 2014 (Schaefer et al., 2014). Since then, several sepsis cases have been reported with ADCs, such as enfortumab vedotin, polatuzumab vedotin, and inotuzumab ozogamicin, in clinical trials (DeAngelo et al., 2020; Sehn et al., 2020; Powles et al., 2021). A pool analysis of clinical trials showed that 28% of cancer patients who received gemtuzumab ozogamicin developed grade 3 to 4 infection, of which 16% progressed to sepsis (Koo and Baden, 2008). However, there are no reviews, meta-analyses, or large cohort studies to identify the association between sepsis and ADCs. The clinical characteristics, broad spectrum, and outcome of sepsis-related toxicities correlated with ADCs remain unknown. Herein, our pharmacovigilance study analyzes the association between ADCs and sepsis-related toxicities using data from the FDA’s Adverse Event Report System (FAERS).

## Materials and methods

### Study design and data sources

The study protocol for our observational, retrospective, cross-sectional pharmacovigilance study of the FAERS database (evaluation of reporting of antibody–drug conjugate-associated sepsis-related toxicities) was registered on ClinicalTrials.gov, NCT05349383. AERS*Mine* (Sarangdhar et al., 2016), a validated web-based platform that analyzes FAERS reports for AE (adverse event) association with drugs, indications, and other features including demographics, reporting period, and report source, was used to conduct this pharmacovigilance analysis. Several high-impact studies (Sarangdhar et al., 2016; Fadini et al., 2018; Fadini et al., 2019; Suarez-Almazor et al., 2019; Sarangdhar et al., 2021) have used AERS*Mine* to analyze FAERS data, including a recent study (van Hasselt et al., 2020) which combined post-marketing data with cell line-derived transcriptomic datasets to identify a gene signature to predict the risk of cardiotoxicity with protein kinase inhibitors. Ethical approval was not required because this study was conducted by using deidentified data.

### Procedures

This study included all sepsis-related toxicities in cancer patients reported between 2004 and 2021 (Q3) and classified by preferred term (PT) under sepsis (SMQ, Standardised MedDRA Query), according to the Medical Dictionary for Regulatory Activities (MedDRA 25.0; Supplementary Material S1 sepsis reports with counts >0 were included). We used case/non-case analysis to study if sepsis was differentially reported with ADCs as compared to other drugs in the complete database. To highlight the underlying association between ADCs and sepsis, we compared the safety signals of sepsis among ADCs and other common cancer regimens, such as chemotherapy, targeted therapy, and their combinations. First, we identified relevant National Comprehensive Cancer Network (NCCN) guidelines (Supplementary Material S1), according to FDA-approved indications of ADCs. Then we extracted different cancer regimens (Supplementary Material S1) from those selected NCCN guidelines. AERS*Mine* was used to analyze sepsis safety signals among different regimens. We used a heatmap to display the landscape of sepsis-related toxicities among anticancer therapies.

For detailed clinical features, we analyzed the sepsis frequencies by age, gender, and different ADCs regimens, and used the forest plot to visualize the difference. The outcome of ADC-related sepsis was also detected. Furthermore, a previous study showed the time-to-onset analysis method does not share the major drawback of disproportionality analysis (DPA) known as the masking effect and could be a complementary tool to detect safety signals apart from traditional DPA (Van Holle et al., 2012). Another study displayed the process of the time-to-onset analysis in detail by using the Weibull distribution (Ando et al., 2019). We detected time to onset of ADC-related sepsis leveraging FAERS raw data in this study.

Drug–drug interaction (DDI) may affect the occurrence and severity of adverse drug reactions. For instance, a higher proportion of patients reported interstitial pneumonitis for nivolumab in combination with epidermal growth factor receptor-tyrosine kinase inhibitors (EGFR-TKIs) vs. treatment with either drug alone (Oshima et al., 2018). Granulocyte colony-stimulating factor (G-CSF) or granulocyte-macrophage colony-stimulating factor (GM-CSF) is usually used to augment myeloid cell functions in cancer patients receiving chemotherapy. Previous research studies showed that granulocyte colony-stimulating factor could enhance the effect of gemtuzumab ozogamicin in acute myeloid leukemia (Leone et al., 2004) and primary prophylaxis with G-CSF may improve outcomes in patients with newly diagnosed stage III/IV Hodgkin lymphoma treated with brentuximab vedotin in addition to chemotherapy (Straus et al., 2020). The expert consensus on the clinical application of antibody–drug conjugates in the treatment of malignant tumors (2020 edition) of China (Professional Committee on Clinical Research of Oncology Drugs CA-CAExpert Committee for Monitoring the Clinical Application of Antitumor DrugsBreast Cancer Expert Committee of National Cancer Quality Control CenterCancer Chemotherapy Quality Control Expert Committee of Beijing Cancer Treatment Quality Control and Improvement Center, 2021) also recommended that colony-stimulating factors could be used to prevent the neutropenia associated with ADCs. So we detected the safety signal of sepsis when colony-stimulating factors were combined with ADCs in the DDI analysis. A previous research study (Zhang et al., 2022) showed that proton pump inhibitors (PPIs) interfere with the antitumor potency of HER2-targeting ADCs due to the inhibition of vacuolar H+-ATPase activity. We inferred that drugs that inhibit gastric acid secretion, such as proton pump inhibitors and H2-receptor antagonists, may alter the risk of sepsis when co-administered with ADCs. Moreover, we searched the DrugBank (Wishart et al., 2018) and found that enfortumab vedotin, brentuximab vedotin, polatuzumab vedotin, tisotumab vedotin, trastuzumab deruxtecan, and loncastuximab tesirine are mainly metabolized by the CYP3A4/5 enzyme. When the activity of the CYP3A4/5 enzyme was affected by other drugs, the metabolism process of ADCs would also be affected. So we also detected the safety signal of sepsis when ADCs were combined with proton pump inhibitors, H2-receptor antagonists, and CYP3A4/5 strong inhibitors.

Since pharmacovigilance studies based on spontaneous reporting systems can be impacted by reporting bias (Noguchi et al., 2021), we further conducted sensitivity analysis by excluding known drugs and indications which may increase susceptibility to sepsis.

### Statistical analysis

In this study, two calculation indicators of disproportionality were used, the reporting odds ratio (ROR) (Rothman et al., 2004) based on the frequentist statistical method and the information component (IC) (Bate et al., 1998) based on the Bayesian statistical method used at the Uppsala Monitoring Centre (UMC). When the lower limit of the 95% credibility interval of ROR (ROR_025_) >1 (Rothman et al., 2004) or the lower limit of the 95% credibility interval of IC (IC_025_) >0 (Bate et al., 1998), significant adverse events were detected. Noren et al. (2013) put forward shrinkage observed-to-expected ratios to provide effective protection against spurious associations in signal detection. This adjustment calculation method was used in our analysis. These IC and ROR are standard pharmacovigilance metrics and have recently been shown to quantitate the spectrum and characteristics of immune checkpoint inhibitor-related cardiovascular toxicity (Salem et al., 2018).

Several methods for detecting DDI have been reported (Noguchi et al., 2019); however, the omega (Ω) shrinkage observed-to-expected ratio measure (Noren et al., 2008; Noguchi et al., 2019) used by the UMC (UMC, 2016) has shown to be the most conservative in DDI signal detection (Noguchi et al., 2020). The detection criterion is the lower limit of the 95% credibility interval of Ω (Ω_025_) >0 (calculation of IC, ROR, and Ω are included in Supplementary Material S1). Safety signals of sepsis-related toxicities among diverse treatment regimens were conducted using the χ2 test (Bonferroni adjustment). All data analyses were performed independently by two or more authors, and all statistical analyses were performed with JMP Pro 16 (SAS Institute Inc., Cary, NC, United States) and Microsoft Excel (2021).

## Results

### ADCs-sepsis disproportionate analysis

**FIGURE 1:**
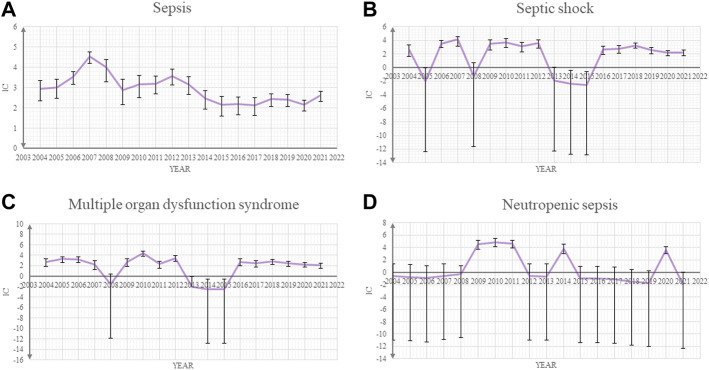
Information component (IC) and its 95% credibility interval over time for **(A)** sepsis, **(B)** septic shock, **(C)** multiple organ dysfunction syndrome, and **(D)** neutropenic sepsis.

Our post-marketing safety signal analysis showed that sepsis and other related toxicities were significantly associated with ADCs. Sepsis (ROR_025_ 6.55 and IC_025_ 2.63), septic shock (ROR_025_ 6.85 and IC_025_ 2.71), multiple organ dysfunction syndrome (ROR_025_ 14.77 and IC_025_ 3.78), neutropenic sepsis (ROR_025_ 16.34 and IC_025_ 3.89), and bacteremia (ROR_025_ 6.49 and IC_025_ 2.59) were the five most common sepsis-related toxicities correlated with ADCs (Table.1). The IC values and their 95% credibility intervals over time for sepsis, septic shock, neutropenic sepsis, and multiple organ dysfunction syndrome, which are top four of the most reported sepsis-related toxicities, are shown in Figure 1.

**TABLE 1:** Sepsis-related toxicities reported with ADC therapy *vs.* the full FAERS database.

	Overall ADCs	Full database	ROR_025_	IC_025_
Total number of ICSRs available	24618	16849672		
Number of ICSRs by sepsis subgroups				
Sepsis	1054	108277	6.55	**2.63**
*Escherichia* bacteremia	25	1848	6.32	2.33
Septic shock	419	38950	6.85	**2.71**
Bacteremia	114	10139	6.49	**2.59**
Systemic inflammatory response syndrome	31	4066	3.69	1.69
Neutropenic sepsis	193	7238	16.34	**3.89**
*Escherichia* sepsis	43	2915	7.58	2.68
*Klebsiella* sepsis	21	1040	9.15	2.68
Staphylococcal sepsis	61	5561	5.9	2.41
Staphylococcal bacteremia	59	3880	8.18	2.84
Enterococcal bacteremia	22	853	11.86	2.97
*Candida* sepsis	14	923	6.21	2.07
Streptococcal bacteremia	20	895	10.03	2.75
Blood culture positive	71	3668	10.69	3.22
Fungal sepsis	13	854	6.11	2.01
Urosepsis	42	8939	2.38	1.13
Multiple organ dysfunction syndrome	408	17778	14.77	**3.78**
Pseudomonal sepsis	62	1708	20.02	3.96
Systemic *candida*	29	1883	7.41	2.56
Fungemia	23	1313	8.07	2.58
Streptococcal sepsis	36	1238	14.71	3.43
Device-related sepsis	19	1803	4.63	1.87
Bacterial sepsis	34	3217	5.21	2.16
Enterococcal sepsis	15	922	6.79	2.2
Biliary sepsis	21	456	21.31	3.47

ADCs, antibody–drug conjugates; FAERS, FDA’s Adverse Event Report System; IC_025_, the lower limit of the 95% credibility interval of information component; ICSR, individual case safety report; ROR_025_, the lower limit of the 95% credibility interval of reporting odds ratio. When IC_025_ > 0 or ROR_025_ > 1, a significant safety signal was detected.

### Clinical features of sepsis-related toxicities during ADC therapies

**FIGURE 2:**
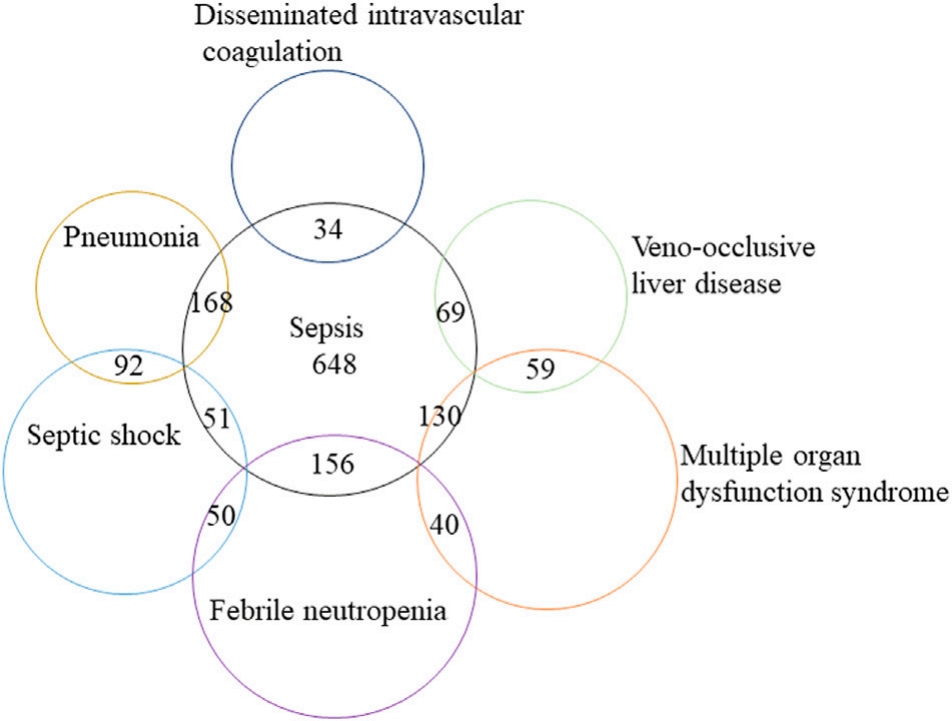
Modified Venn diagram showing the overlap between distinct classes of sepsis-related toxicities and other lethal AEs, such as veno-occlusive liver disease. AEs, adverse events.

**FIGURE 3:**
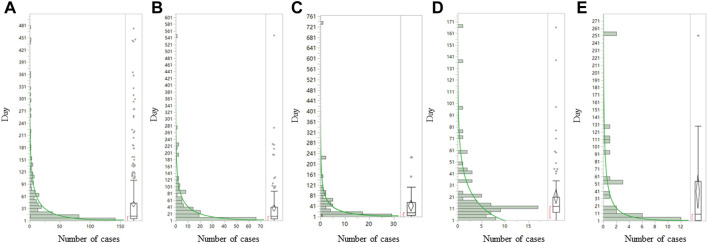
Time-to-onset analysis for **(A)** sepsis, **(B)** septic shock, **(C)** multiple organ dysfunction syndrome, **(D)** neutropenic sepsis, and **(E)** bacteremia associated with ADCs. ADCs, antibody–drug conjugates. The *Y* axis is days to onset and the *X* axis is the number of case *(N)*. The left side of each graph is a histogram and the right side is a box-and-whisker diagram with outlies. The green line on the histogram was drawn by fitting the Weibull distribution.

We further analyzed the clinical characteristics of the five most common sepsis-related toxicities correlated with ADCs (Table 2). About 71.1% (1,556/2,188) of cases were reported by medical professionals, and 48.7% (1,065/2,188) of cases were reported in 2019–2021. ADC-related sepsis cases were predominantly reported largely in patients with acute myeloid leukemia (39.9% of all cases; n = 973/2,441). The co-reported toxicities’ landscape among sepsis-related toxicities shows that sepsis, septic shock, and multiple organ dysfunction syndrome not only overlap with each other but also with other serious toxicities such as veno-occlusive liver disease (Figure 2). To evaluate the onset of ADC-induced sepsis, we conducted time-to-onset analysis using a curated FAERS dataset (Khaleel et al., 2022). The β-coefficient and its 95% CI for sepsis, septic shock, and bacteremia were less than one, suggesting that the onset time of ADC-induced sepsis is the early failure type, and approximately 60% of sepsis, septic shock, and bacteremia due to ADC therapies developed within 26.6–32.3 days. However, the β and 95% CI of neutropenic sepsis include 1, and nearly 60% of patients who received ADCs would develop neutropenic sepsis within 3 weeks (Table 3; Figure 3).

**TABLE 2:** Clinical characteristics of sepsis-related toxicities correlated with ADCs.

Characteristics	Sepsis	Septic shock	Multiple organ dysfunction syndrome	Neutropenic sepsis	Bacteremia
	N (%)	N (%)	N (%)	N (%)	N (%)
Total number of reporting source	1,054	419	408	193	114
Medical staff	742 (70.4)	306 (73.0)	281 (68.9)	151 (78.2)	76 (66.7)
Non-medical staff	312 (29.6)	113 (27.0)	127 (31.1)	42 (21.8)	38 (33.3)
Reporting year					
2016–2021 (Q3)	511 (48.5)	230 (54.9)	189 (46.3)	80 (41.5)	55 (48.2)
2010–2015	236 (22.4)	86 (20.5)	112 (27.5)	79 (40.9)	29 (25.4)
2004–2009	307 (29.1)	103 (24.6)	107 (26.2)	34 (17.6)	30 (26.3)
Gender					
Male	496 (54.3)	215 (58.6)	211 (59.4)	94 (54.0)	52 (52.0)
Female	418 (45.7)	152 (41.4)	144 (40.6)	80 (46.0)	48 (48.0)
Data available	914	367	355	174	100
Age-group, years					
0–14	26 (3.6)	1 (0.3)	22 (6.8)	3 (2.0)	1 (1.1)
15–65	409 (56.5)	232 (71.8)	193 (59.6)	106 (70.2)	60 (66.7)
>=66	289 (39.9)	90 (27.9)	109 (33.6)	42 (27.8)	29 (32.2)
Data available	724	323	324	151	90
Drugs (different payloads)					
Tubulin polymerization inhibitors	442 (42.0)	203 (48.5)	145 (35.5)	78 (40.4)	47 (41.2)
Trastuzumab emtansine	89 (20.1)	19 (9.4)	15 (10.3)	32 (41.0)	8 (17.0)
Enfortumab vedotin	5 (1.1)	2 (1.0)	8 (5.5)	0	2 (4.3)
Brentuximab vedotin	297 (67.3)	157 (77.3)	113 (78.0)	36 (46.2)	17 (36.2)
Polatuzumab vedotin	35 (7.9)	21 (10.3)	7 (4.8)	8 (10.3)	15 (31.9)
Belantamab mafodotin	16 (3.6)	4 (2.0)	2 (1.4)	2 (2.5)	5 (10.6)
DNA-damaging agents	612 (58.0)	216 (51.5)	263 (64.5)	115 (59.6)	67 (58.8)
Gemtuzumab ozogamicin	501 (81.9)	186 (86.1)	214 (81.4)	85 (73.9)	52 (77.6)
Inotuzumab ozogamicin	91 (14.9)	27 (12.5)	49 (18.6)	30 (26.1)	15 (22.4)
Trastuzumab deruxtecan	6 (1.0)	0	0	0	0
Sacituzumab govitecan	14 (2.2)	3 (1.4)	0	0	0
Indications					
Breast cancer	96 (9.1)	10 (2.4)	10 (2.5)	21 (10.9)	0
Non-Hodgkin’s lymphoma	0	0	0	12 (6.2)	0
Hodgkin’s disease	139 (13.2)	71 (16.9)	64 (15.7)	21 (10.9)	0
Diffuse large b-cell lymphoma	46 (4.4)	15 (3.6)	0	0	0
T-cell lymphoma	41 (3.9)	11 (2.6)	10 (2.5)	0	0
Anaplastic large-cell lymphoma	15 (1.4)	0	0	0	0
Acute myeloid leukemia	426 (40.4)	148 (35.3)	169 (41.4)	65 (33.7)	38 (33.3))
Acute lymphocytic leukemia	62 (5.9)	21 (5.0)	34 (8.3)	11 (5.7)	0
Plasma cell myeloma	12 (1.1)	0	0	0	0
Concurrent symptoms/syndromes					
Sepsis	N/A	51 (12.2)	130 (31.9)	0	0
Septic shock	51 (4.8)	N/A	102 (25.0)	0	0
Multiple organ dysfunction syndrome	130 (12.3)	102 (24.3)	N/A	36 (18.7)	0
Neutropenic sepsis	0	0	22 (5.4)	N/A	0
Bacteremia	0	0	0	0	N/A
Other co-reported AEs					
Febrile neutropenia	156 (14.8)	50 (11.9)	40 (4.8)	0	26 (22.8)
Veno-occlusive liver disease	69 (6.6)	0	59 (14.5)	15 (7.8)	13 (11.4)
Pneumonia	168 (15.9)	92 (22.0)	0	17 (8.8)	0
Acute respiratory distress syndrome	35 (3.3)	33 (7.9)	0	0	0
Disseminated intravascular coagulation	34 (3.3)	0	0	0	0

ADCs, antibody–drug conjugates; N/A, not applicable; AEs, adverse events.

**TABLE 3:** Time-to-onset analysis of ADC-induced sepsis and related toxicities in the FAERS database.

Adverse event	Case (N)	α (95% CI)	β (95% CI)
Sepsis	405	31.2 (26.8–36.2)	0.69 (0.42–0.74)
Septic shock	159	30.1 (24.0–37.6)	0.74 (0.65–0.82)
Neutropenic sepsis	81	21.0 (16.4–26.7)	0.96 (0.81–1.11)
Multiple organ dysfunction syndrome	77	32.3 (22.7–45.3)	0.69 (0.58–0.81)
Bacteremia	34	26.6 (14.3–47.8)	0.61 (0.46–0.78)

The Weibull distribution is a continuous probability distribution used to analyze life data, model failure times, and access product reliability, which also could be used to conduct time-to-onset in pharmacovigilance. N, we included available data which contain the event date and ADCs therapy start date. α, scale parameter, could be used to express time-to-onset duration. β, shape parameter, could be used to confirm the distribution type: early failure type (β<1), random failure type (95% CI of β include 1), and wear-out type (β>1). 95% CI, 95% credibility interval.

**FIGURE 4:**
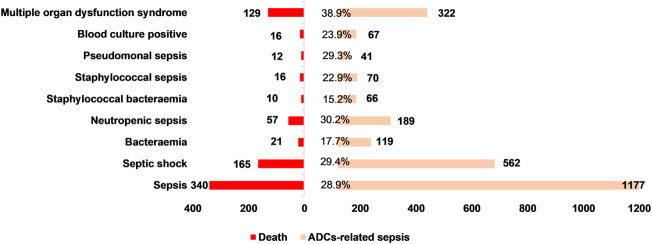
Death cases and their proportion in ADC-related sepsis.

**FIGURE 5:**
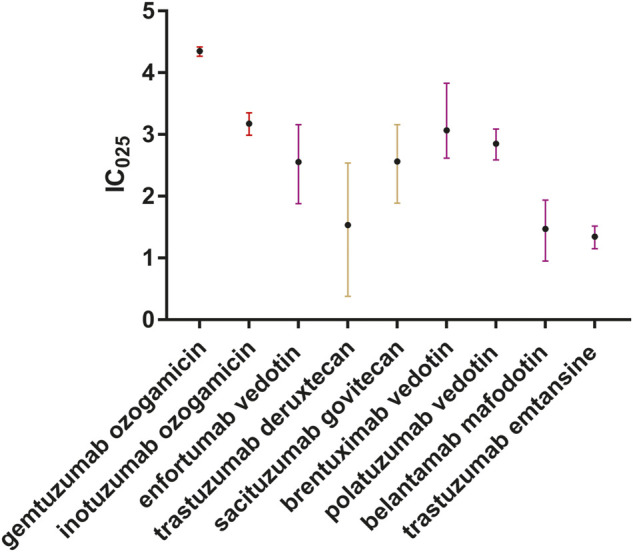
Forest plot of IC_025_ values of ADC-related sepsis. We included all sepsis-related toxicities correlated with ADCs. Different colors stand for different payloads. Red indicates calicheamicin, brown indicates DNA-damaging agents, and purple indicates tubulin polymerization inhibitors.

**FIGURE 6:**
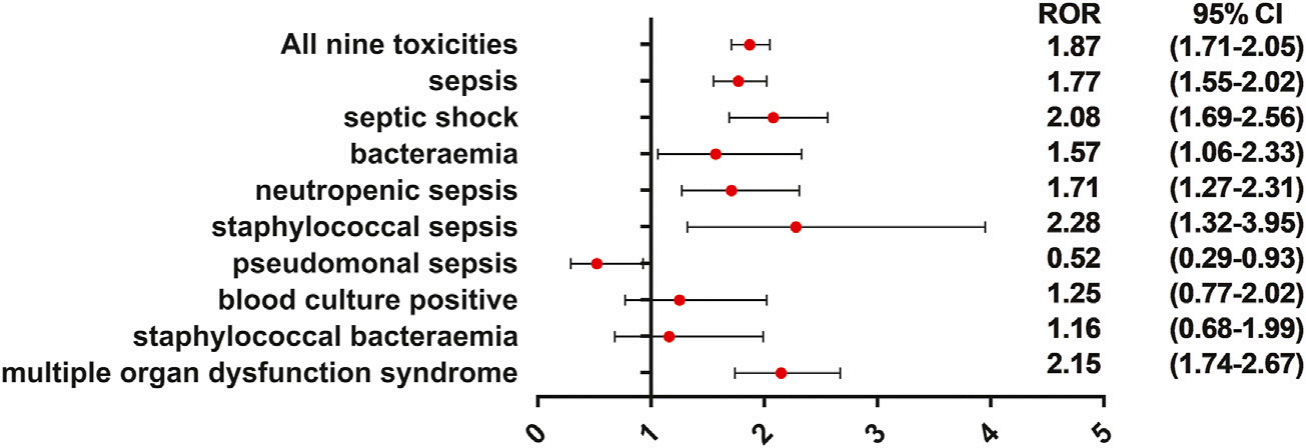
ROR of sepsis-related toxicities when males vs. females. This forest plot showed that sepsis, septic shock, bacteremia, neutropenic sepsis, staphylococcal sepsis, and multiple organ dysfunction syndrome were significantly more reported with males. This may provide alert of sepsis for clinicians when they are using ADC therapies to treat male cancer patients.

We further identified that death, as an outcome, was common in patients with ADC-related sepsis. We identified that 340 of 1,177 (28.9%), 165 of 562 (29.4%), 129 of 322 (40.1%), 57 of 189 (30.1%), and 21 of 119 (17.7%) death cases were reported in patients who developed sepsis, septic shock, multiple organ dysfunction syndrome, neutropenic sepsis, and bacteremia, respectively (Figure 4). We conducted the subgroup analysis of sepsis-related toxicities according to ADC categories, gender, and age. Subgroup analysis of sepsis-related toxicities stratified by ADC categories, gender, and age revealed that both gemtuzumab ozogamicin and inotuzumab ozogamicin, with calicheamicin payload, showed higher safety concerns for sepsis than any other ADCs (Figure 5; Table 4). Males showed significantly higher safety concern for sepsis-related events than females (Figure 6, *p* <0.0001). There was no significant difference for sepsis-related toxicities among different age-groups (0–14 years, 15–24 years, 25–65 years, or >65 years, *p* >0.05).

**TABLE 4:** Safety signals among different ADC drugs (vs. other drugs in the full database).

Drug name	All AEs	Targeted AEs	ROR (95% CI)	IC (95% CI)
Gemtuzumab ozogamicin	4,923	1,389	28.52 (26.80–30.35)	4.36 (4.27–4.42)
Inotuzumab ozogamicin	1,596	167	10.53 (9.22–12.01)	3.19 (2.99–3.35)
Enfortumab vedotin	191	14	7.80 (4.92–12.37)	2.63 (1.88–3.16)
Trastuzumab deruxtecan	129	8	3.98 (1.86–8.51)	1.69 (0.38–2.54)
Sacituzumab govitecan	178	19	7.84 (4.94–12.44)	2.64 (1.89–3.16)
Brentuximab vedotin	7,632	543	7.33 (6.80–7.90)	2.75 (2.62–2.83)
Polatuzumab vedotin	1,196	114	8.30 (6.92–9.95)	2.88 (2.59–3.09)
Belantamab mafodotin	457	24	3.05 (2.15–4.32)	1.53 (0.95–1.94)
Trastuzumab emtansine	4,841	106	2.65 (2.33–3.01)	1.37 (1.15–1.52)

ADCs, antibody–drug conjugates; AEs, adverse events; IC, information component; and ROR, reporting odds ratio. 95% CI, 95% credibility interval.

### Sepsis signals in ADCs and other anticancer regimens

**FIGURE 7:**
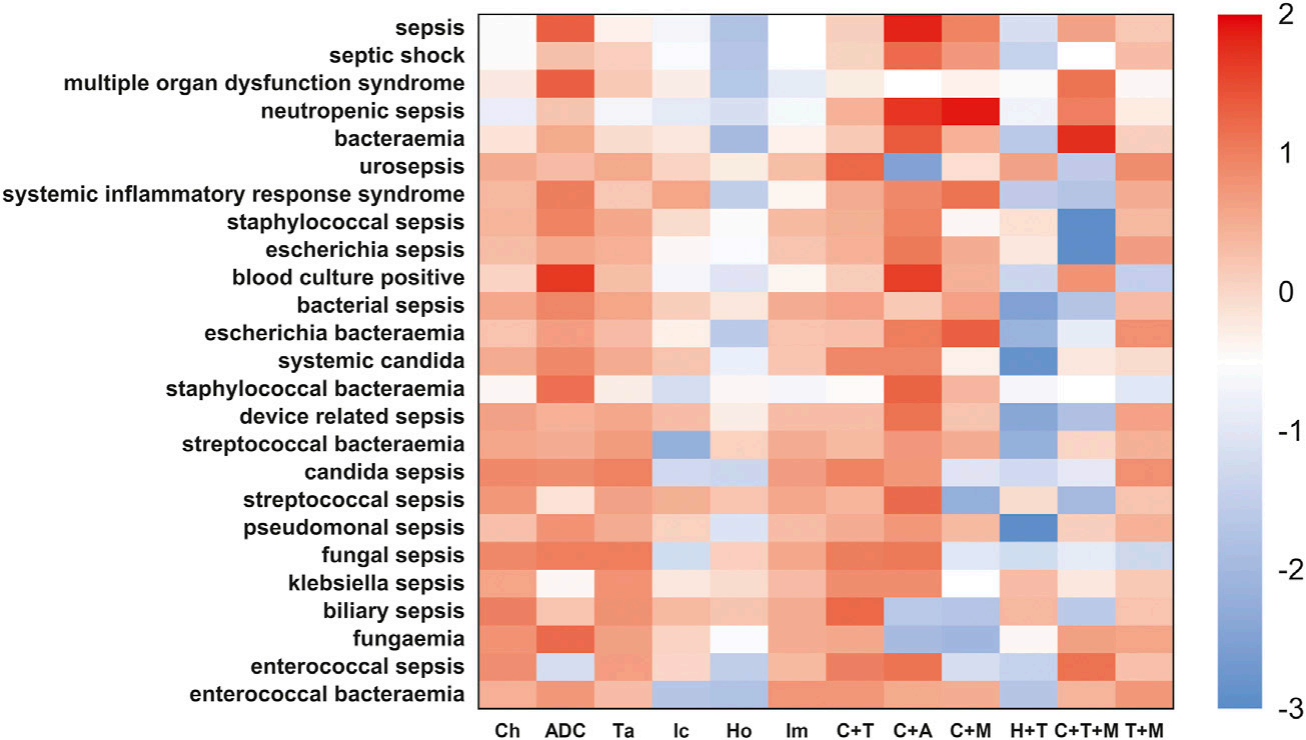
Sepsis-related toxicities landscape among ADCs and other anticancer therapies (vs. global control). The heatmap represents a comparative analysis of differential risk profiles of sepsis and related AEs across cancer drug regimens, such as Ch-chemotherapy, ADC-antibody–drug conjugates, Ta-targeted therapy, Ic-immunotherapy, HO-endocrine therapy, Im-immunomodulatory drugs, and C+T-chemotherapy combined with targeted therapy. These regimens were extracted from guidelines of FDA-approved indications for ADCs. For the more conservative global controls, we selected cancer patients not on any of the aforementioned cancer regimens, that is, patients not taking Ic, Ta, Ch, Ic+Ta, Ch+Ic, and Ch+Ta. The red color indicates a high risk of adverse effect in different cancer drug regimens. This analysis demonstrated ADCs had the highest safety concern for sepsis, multiple organ dysfunction syndrome, pseudomonal sepsis, fungemia, and blood culture positive than any other cancer drug regimens (*p* <0.05).

**FIGURE 8:**
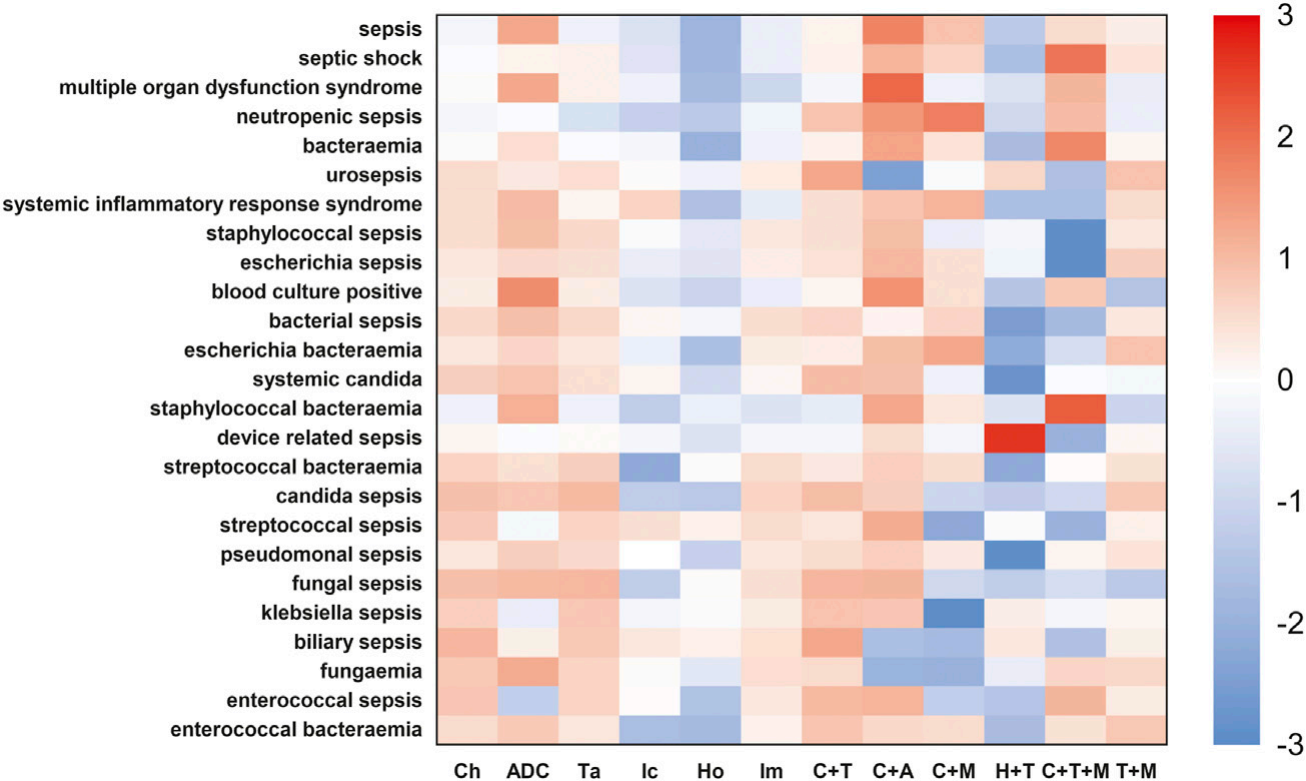
Sepsis-related toxicities landscape among ADCs and other anticancer therapies (vs. in-class control). In-class control represented other cancer patients not on a specific drug class, for instance, the controls for the Ic group were cancer patients not on Ic therapy. Similarly, controls for Ta were cancer patients not taking any targeted therapy. The red color indicates a high risk of adverse effect in different cancer drug regimens. This analysis using another control group confirmed the findings in Figure 7 that ADCs had the highest safety concern for sepsis, multiple organ dysfunction syndrome, pseudomonal sepsis, fungemia, and blood culture positive among any other cancer drug regimens (*p* <0.05).

We further compared the incidence of sepsis-related toxicities across different cancer regimens (Figure 7, Figure 8). When compared with the global controls (all cancer patients) or within class (e.g., ADCs, targeted therapy, and immunotherapy), we found that ADCs presented the highest safety concern for sepsis, multiple organ dysfunction syndrome, pseudomonal sepsis, fungemia, and blood culture positive compared with any other cancer drug regimens (*p* <0.05). We also noted that the combination of ADCs and chemotherapy significantly increased the safety concern of septic shock, neutropenic sepsis, and bacteremia (Supplementary Material S3).

### Co-administration and drug–drug interaction signal analysis

First, we analyzed the safety signal for sepsis when ADCs were co-administrated with the granulocyte colony-stimulating factor (G-CSF) or granulocyte-macrophage colony-stimulating factor (GM-CSF), proton pump inhibitors, H2-receptor antagonists, and CYP3A4/5 strong inhibitors. ADCs, when combined with colony-stimulating factors (G-CSF/GM-CSF), have a comparable safety signal (IC_025_, 2.58 vs. 2.63) for sepsis than ADCs’ monotherapy (Table 5). We analyzed the safety profiles for the combination therapy of ADCs with PPIs or H2-receptor antagonists. Sepsis (IC_025_ 2.75), septic shock (IC_025_ 2.48), neutropenic sepsis (IC_025_ 3.44), multiple organ dysfunction syndrome (IC_025_ 3.26), pseudomonal sepsis (IC_025_ 4.66), biliary sepsis (IC_025_ 4.25), and streptococcal sepsis (IC_025_ 3.32) were significantly associated with ADC and PPI combination therapy (Table 6). Safety concerns for sepsis (IC_025_ 3.66) and septic shock (IC_025_ 2.38) were detected for the combination of ADCs and H2-receptor antagonists (Table 7). We then included the most common CYP3A4/5 strong inhibitors which contain protease inhibitors, imidazole and triazole derivatives, and macrolides, and analyzed sepsis-related toxicities when ADCs were combined with the previously listed CYP3A4/5 inhibitors. We identified that risk for sepsis (ROR_025_ 11.57 and IC_025_ 3.06) and septic shock (ROR_025_ 13.91 and IC_025_ 3.95) was significantly higher for the combination than for ADC monotherapy (Table 8).

**TABLE 5:** Safety signals for sepsis-related toxicities reported with ADCs and colony-stimulating factors (G-CSF/GM-CSF) combination therapy *vs.* the full FAERS database.

	Overall AEs of ADCs+G-CSF/GM-CSF	Full database	ROR_025_	IC_025_
Total number of ICSRs available	1914	16,849,672	6.65	2.58
Number of ICSRs by sepsis-related AE subgroups				
Sepsis	96	108277		
Multiple organ dysfunction syndrome	41	17778	15.24	3.52
Septic shock	56	38950	9.98	3.08
Neutropenic sepsis	36	7238	32.21	4.23

ADCs, antibody–drug conjugates; G-CSF, granulocyte colony-stimulating factor; GM-CSF, granulocyte-macrophage colony-stimulating factor; and AEs, adverse events.

**TABLE 6:** Sepsis safety signals for ADCs and proton pump inhibitors combination therapy.

	Overall AEs of ADCs+proton pump inhibitors	Full database	ROR_025_	IC_025_
Total number of ICSRs available	3798	16,849,672	7.34	2.75
Number of ICSRs by sepsis-related AE subgroups				
Sepsis	197	1,08,277		
Septic shock	68	38,950	6.20	2.48
Neutropenic sepsis	34	7,238	25.05	3.44
Multiple organ dysfunction syndrome	58	17,778	11.36	3.26
Pseudomonal sepsis	33	1,708	62.36	4.66
Biliary sepsis	19	456	122.30	4.25
Streptococcal sepsis	14	1,238	30.04	3.32

**TABLE 7:** Sepsis safety signals for ADC and H2-receptor antagonists combination therapy.

	Overall AEs of ADCs+H2-receptor antagonists	Full database	ROR_025_	IC_025_
Total number of ICSRs available	1251	16,849,672	15.11	3.66
Number of ICSRs by sepsis-related AE subgroups				
Sepsis	131	108277		
Septic shock	27	38950	6.51	2.38

**TABLE 8:** Sepsis safety signals for ADCs and CYP3A4/5 strong inhibitors combination therapy.

	Overall AEs of ADCs + CYP3A4/5 inhibitors	Full database	ROR_025_	IC_025_
Total number of ICSRs available	341	16,849,672	11.57	3.06
Number of ICSRs by sepsis-related AE subgroups				
Sepsis	33	108277		
Septic shock	17	38950	13.91	3.95

CYP3A4/5 strong inhibitors; we included protease inhibitors, imidazole and triazole derivatives, and macrolides in this study.

Second, we analyzed the drug–drug interaction signals for sepsis between ADCs and granulocyte colony-stimulating factor (G-CSF) or granulocyte-macrophage colony-stimulating factor (GM-CSF), proton pump inhibitors, H2-receptor antagonists, and CYP3A4/5 strong inhibitors. We did not detect other drug interaction signals for sepsis-related toxicities, except for *Escherichia* bacteremia (Ω_025_ 0.37), for the ADCs-G-CSF/GM-CSF combination. We further identified drug interaction signals for ADCs–PPIs combination therapy and found increased safety concerns for enterococcal bacteremia (Ω_025_ 0.34), systemic inflammatory response syndrome (Ω_025_ 0.09), pseudomonal sepsis (Ω_025_ 1.59), streptococcal sepsis (Ω_025_ 0.95), and biliary sepsis (Ω_025_ 3.24). In contrast, DDI signals were detected for sepsis (Ω_025_ 0.79) and bacteremia (Ω_025_ 0.43) when ADCs were combined with H2-receptor antagonists. DDI signals were detected for sepsis (Ω_025_ 0.28) and fungemia (Ω_025_ 0.02) for the combination of ADCs and CYP3A4/5 inhibitors (Table 9) (Supplementary Material S2).

**TABLE 9:** Drug–drug interaction analysis for ADCs and other drugs.

Drug 1	Drug 2	Adverse effect	Ω_025_
ADCs	Colony-stimulating factors	*Escherichia* bacteremia	0.37
ADCs	Proton pump inhibitors	Enterococcal bacteremia	0.34
ADCs	Proton pump inhibitors	Systemic inflammatory response syndrome	0.09
ADCs	Proton pump inhibitors	Pseudomonal sepsis	1.59
ADCs	Proton pump inhibitors	Streptococcal sepsis	0.95
ADCs	Proton pump inhibitors	Biliary sepsis	3.24
ADCs	H2-receptor antagonists	Sepsis	0.79
ADCs	H2-receptor antagonists	Bacteremia	0.43
ADCs	H2-receptor antagonists	Streptococcal bacteremia	0.09
ADCs	H2-receptor antagonists	Procalcitonin increased	0.41
ADCs	H2-receptor antagonists	*Serratia* sepsis	1.59
ADCs	H2-receptor antagonists	*Salmonella* bacteremia	1.05
ADCs	CYP3A4/5 strong inhibitors	Sepsis	0.28
ADCs	CYP3A4/5 strong inhibitors	Fungemia	0.02

Ω_025,_ the lower limit of the 95% credibility interval of shrinkage observed-to-expected ratio. When Ω_025_ > 0, a significant drug–drug interaction signal was detected.

### Sensitivity analysis

Some confounding factors such as indications and other known drug reactions may affect the safety signals of ADC-related toxicities. We excluded diseases (autologous hematopoietic stem cell transplant, allogenic stem cell transplantation, diabetes, organ transplant, chronic obstructive pulmonary disease, alcohol abuse, indwelling catheter management, surgery, and HIV infection) as sepsis may occur preferentially in patients with these conditions. We also excluded known drug reactions of sepsis (extracted from FDA’s labels) and chose the role code as “primary suspect drug.” After adjusting for confounders, no significant change was observed in the safety signals (Table 10).

**TABLE 10:** Safety signals for sepsis-related toxicities reported with ADC therapy *vs.* the full FAERS database after sensitivity analysis.

	Overall ADCs	Full database	ROR_025_	IC_025_
Total number of ICSRs available	12501	16,849,672		
Number of ICSRs by sepsis-related AE subgroups				
Sepsis	650	1,08,277	7.88	2.88
Multiple organ dysfunction syndrome	232	17,778	15.90	3.87
Septic shock	212	38,950	6.53	2.63
Blood culture positive	50	3,668	14.13	3.50
Bacteremia	65	10,139	6.84	2.62
Neutropenic sepsis	95	7,238	14.72	3.68
Staphylococcal sepsis	39	5,561	6.96	2.56
Staphylococcal bacteremia	38	3,880	9.70	2.97
Bacterial sepsis	21	3,217	5.77	2.17
*Escherichia* sepsis	28	2,915	9.02	2.79
Pseudomonal sepsis	32	1,708	18.15	3.61
Systemic *candida*	22	1,883	10.47	2.85
Systemic inflammatory response syndrome	17	4,066	3.51	1.50
Fungemia	15	1,313	9.36	2.52
Enterococcal bacteremia	12	853	10.88	2.49

## Discussion

To the best of our knowledge, this is the first large-scale study to identify the association between ADCs and sepsis. Meanwhile, the first case of sepsis (Schaefer et al., 2014) correlated with brentuximab vedotin was reported in 2014, and there have been no reviews, meta-analyses, or retrospective studies focusing on the underlying association between ADCs and sepsis. This study is the first effort to systematically associate sepsis-related toxicities occurrence with ADCs and characterize a large population of affected patients. The novelty and significance of our study is summarized in three aspects:

First, we analyzed the significant association between ADCs and sepsis, and further uncovered the clinical features of ADC-related sepsis. Through disproportionality analysis of the FAERS database, we detected significantly high safety concern for sepsis and related toxicities (including septic shock, multiple organ dysfunction syndrome, neutropenic sepsis, and bacteremia) in ADCs compared to other drugs. Safety profiles for sepsis, septic shock, and multiple organ failure syndrome have consolidated in the recent years without significant fluctuations year on year. Although spontaneous reporting systems are subject to various reporting biases which may impact signal scores, in this study, signal scores remained stable across the study years. Certainly, we cannot validate this beyond the limitations of the spontaneous reporting system, but the stable safety signal may enhance the validity of the signal. The sepsis-related toxicities that correlated with ADCs not only overlapped with each other but also with other serious toxicities such as veno-occlusive liver disease. A previous study (Kim et al., 2019) showed that patients with chemotherapy-induced febrile neutropenia are vulnerable to extended-spectrum b-lactamase-producing Enterobacteriaceae infection, which is prone to cause septic shock. We also detected the overlap of febrile neutropenia with sepsis or septic shock. The time-to-onset information of ADC-related sepsis is scarce in the published literature or FDA’s drug labels. We fitted a Weibull distribution to estimate the duration between ADC administration and sepsis occurrence. The results of Weibull parameter α and β values of ADC-related sepsis suggested that sepsis, septic shock, and bacteremia occurred within a month and classified into the early failure type, while neutropenic sepsis classified into the random failure type. Clinicians should be vigilant in the early recognition and prevention of this kind of toxicity. Different ADCs are constituted of different cytotoxic payloads and targeted monoclonal antibody. A recent review (Lievano et al., 2021) indicated that key toxicities for ADCs are primarily associated with off-target effects from the payload. We found that gemtuzumab ozogamicin and inotuzumab ozogamicin presented higher safety concerns for sepsis than any other kinds of ADCs, which may indicate the role of calicheamicin in the elevated risk of sepsis than other kinds of payloads. Males showed significantly higher safety concern for sepsis than females; however, age did not correlate with ADC-related sepsis. We noticed that the first case of sepsis associated with ADCs is male (Schaefer et al., 2014), and another pharmacovigilance study related to cutaneous toxicity associated with enfortumab vedotin (Yang et al., 2021) indicated that most cases were male (76.42%). This is in line with our study. But another study (Li et al., 2022) focusing on arrhythmia association with antibody–drug conjugates showed that gender differences among affected patients are not significant (female vs. male = 43.57 vs. 42.86%). The aforementioned result showed that gender difference is not the same in different adverse events of ADCs. The International Conference on Harmonization considers older people a “special population” as they differ from younger adults in terms of comorbidity, polypharmacy, pharmacokinetics, and greater vulnerability to adverse drug reactions (Davies and O'Mahony, 2015). However, another research study (Begaud et al., 2002) analyzed 92,043 spontaneous domestic reports in the French pharmacovigilance database and argued the main factor for the risk of adverse drug reaction is the number of drug treatments and not the age itself. Further research needs to be conducted to explore the influence of gender and age on the risk of ADC-related sepsis. We identified that 766 of 2613 (29.3%) cases who developed ADC-related sepsis or related toxicity died during therapy, which reflected a disproportional mortal rate.

Second, we identified a significantly high safety concern of sepsis and multiple organ dysfunction syndrome associated with ADCs compared to other anticancer drug therapies within FDA-approved indications for ADCs. We have not found guidelines related to toxicity management of ADCs. The FDA’s drug labels that indicate serious infections and opportunistic infections are likely adverse reactions of brentuximab vedotin. No sepsis-related toxicities were mentioned in ADCs’ labels. In contrast, our pharmacovigilance study indicates that ADCs present the strongest safety concern for sepsis and multiple organ dysfunction syndrome among all included anticancer therapies, except for ADCs combined with chemotherapy.

Third, we detected drug interaction signals and found an increased risk of sepsis when ADCs were co-administrated with colony-stimulating factors, proton pump inhibitors, H2-receptor antagonists, and CYP3A4/5 strong inhibitors. A previous meta-analysis (Bo et al., 2011) indicated that there is no current evidence supporting the routine use of G-CSF or GM-CSF in patients with sepsis, and G-CSF or GM-CSF could not increase the reversal rate from infection in patients with sepsis. Our pharmacovigilance analysis also detected significant risk of sepsis in cancer patients who received ADCs and colony-stimulating factors (G-CSF/GM-CSF) (IC_025_=2.58), which further confirmed that G-CSF or GM-CSF could not increase the reversal rate from infection in patients with sepsis when co-administered with ADCs. We also detected DDI signals between ADCs and gastric medications, such as proton pump inhibitors and H2-receptor antagonists, for several subtypes of sepsis. Since most of the ADCs are metabolized by the CYP3A4/5 enzyme, the safety signal for sepsis was elevated when ADCs were co-administrated with CYP3A4/5 strong inhibitors. This result demonstrates that physicians need to be vigilant when ADCs are co-administrated with the aforementioned medications.

In summary, we detected a significant safety concern for ADC-related sepsis in cancer patients. The clinical features and drug interaction signals were explored. Further studies are warranted to describe underlying mechanisms and develop preventive measures of ADC-related sepsis.

### Limitations

There are several limitations to this study. First, adverse event reports come from heterogeneous sources, which raise the possibility of incomplete information. Second, detailed clinical information is unavailable from the FAERS database, thus limiting our quality assessment to those reports. Third, we could not definitively confirm the incidence of events using spontaneous reporting systems but only for hypotheses generation. Fourth, we could not combine data from randomized controlled trails with the FAERS database because sepsis cases are rare in ADCs’ trials. Fifth, in the time-to-onset analysis, the Weibull distribution does not incorporate the effects of concomitant medications. Sixth, *underreporting* bias is an intrinsic limitation in research using a spontaneous database. Our time trends analysis for IC and credibility intervals in Figure 1 show some peaks and small differences through time, which can be partially explained by differences in reporting rates. However, cases in the FAERS database cover many countries in the world, thus ensuring an unparalleled global assessment of ADC-related sepsis in diverse real-world clinical settings.

## Conclusion

Antibody–drug conjugates are promising and cutting-edge anticancer therapies which significantly improve the refractory tumor response and render patients with increased survival. However, severe sepsis-related toxicities are significantly associated with ADCs compared to other common cancer drug therapies. Patients on gemtuzumab ozogamicin and inotuzumab ozogamicin are more prone to develop sepsis than with other ADCs. In this study, males showed a significantly higher safety concern for sepsis than females, while age did not influence the safety signal of ADC-related sepsis. We identified that 766 of 2,613 (29.3%) patients who developed ADC-related sepsis died during treatment. Sepsis, septic shock, multiple organ dysfunction syndrome, and bacteremia tend to occur in the early stage after ADCs’ administration (within a month). G-CSF/GM-CSF, proton pump inhibitors, H2-receptor antagonists, and CYP3A4/5 inhibitors may synergistically increase the risk of sepsis with ADCs. Further studies need to be conducted to uncover the mechanism of sepsis correlated with ADCs. Physicians should be aware of the safety concern of sepsis, and take early recognition and prevention measures when they are treating cancer patients with ADCs.

## Data Availability

The original contributions presented in the study are included in the article/Supplementary Material; further inquiries can be directed to the corresponding author.
