# Supplementary material for: Do antibody–drug conjugates increase the risk of sepsis in cancer patients? A pharmacovigilance study
Source: Front Pharmacol. 2022 Nov 9;13:967017. doi: 10.3389/fphar.2022.967017 (PMC9710632; doi:10.3389/fphar.2022.967017)
Supplement: Supplementary file 1 [file DataSheet3.zip › AERSMine load files/AERSMine load files Name.docx]

**AERS*Mine* load files Name**

**Note:** This study used data from AERS*Mine,* a web-based platform to visit curated FAERS data. We would like to upload some load files (for searching and repeatability of raw data) for editors and reviewers to review. Those files with a suffix: .aers. The followings are their name. Kindly contact corresponding author if you have any questions related to raw data of this study.

1.Sepsis (SMQ) correlated with ADCs

2.Most common sepsis-related toxicities correlated with ADCs

3.Sepsis correlated with ADCs and other anticancer therapies

4.Sensitivity analysis

5.Sepsis safety signal of ADC and colony stimulating factors combination

6.DDI analysis between ADCs and colony stimulating factors

7.Sepsis signal for ADC and CYP3A4/5 inhibitors combination

8.DDI analysis between ADCs and CYP3A4/5 strong inhibitors

9.Safety signal between ADCs and h2-receptor antagonists

10.DDI analysis between ADCs and h2-receptor antagonists

11.Safety signal of sepsis for ADCs and PPIs combination

12.DDI analysis between ADCs and PPIs combination
